# Analysis of Online Peripartum Depression Communities: Application of Multilabel Text Classification Techniques to Inform Digitally-Mediated Prevention and Management

**DOI:** 10.3389/fdgth.2021.653769

**Published:** 2021-05-21

**Authors:** Alexandra Zingg, Tavleen Singh, Sahiti Myneni

**Affiliations:** School of Biomedical Informatics, University of Texas Health Science Center at Houston, Houston, TX, United States

**Keywords:** digital health, peripartum, mental health, machine learning, social media

## Abstract

Peripartum depression (PPD) is a significant public health problem, yet many women who experience PPD do not receive adequate treatment. In many cases, this is due to social stigmas surrounding PPD that prevent women from disclosing their symptoms to their providers. Examples of these are fear of being labeled a “bad mother,” or having misinformed expectations regarding motherhood. Online forums dedicated to PPD can provide a practical setting where women can better manage their mental health in the peripartum period. Data from such forums can be systematically analyzed to understand the technology and information needs of women experiencing PPD. However, deeper insights are needed on how best to translate information derived from online forum data into digital health features. In this study, we aim to adapt a digital health development framework, *Digilego*, toward translation of our results from social media analysis to inform digital features of a mobile intervention that promotes PPD prevention and self-management. The first step in our adaption was to conduct a user need analysis through semi-automated analysis of peer interactions in two highly popular PPD online forums: What to Expect and BabyCenter. This included the development of a machine learning pipeline that allowed us to automatically classify user post content according to major communication themes that manifested in the forums. This was followed by mapping the results of our user needs analysis to existing behavior change and engagement optimization models. Our analysis has revealed major themes being discussed by users of these online forums- family and friends, medications, symptom disclosure, breastfeeding, and social support in the peripartum period. Our results indicate that Random Forest was the best performing model in automatic text classification of user posts, when compared to Support Vector Machine, and Logistic Regression models. Computerized text analysis revealed that posts had an average length of 94 words, and had a balance between positive and negative emotions. Our Digilego-powered theory mapping also indicated that digital platforms dedicated to PPD prevention and management should contain features ranging from educational content on practical aspects of the peripartum period to inclusion of collaborative care processes that support shared decision making, as well as forum moderation strategies to address issues with cyberbullying.

## Introduction

Peripartum depression (PPD) is a condition which affects ~1 in 10 pregnant women and new mothers in the U.S. every year (1, 2). It is attributed to a variety of factors, ranging from the biological fluctuation of hormones in the peripartum period to socioeconomic factors such as, the additional economic strain that may come with having a new infant (3). PPD symptoms include: changes in energy levels, change in appetite, feelings of guilt or worthlessness, inability to bond with the infant, and thoughts of harming oneself or the infant (4). Some risk factors for PPD are: a previous history of depression, complications during pregnancy, or after childbirth, low socioeconomic status, multiparity (a birth resulting in two or more children), and adverse life events (5). PPD can be treated with therapy programs such as cognitive behavioral therapy, and/or pharmacotherapy. The most common medications of choice for PPD are those of the selective serotonin-reuptake inhibitors (SSRIs) class (6, 7). If untreated, PPD can result in adverse health outcomes such as longer depression episodes for women and later cognitive and behavioral problems for the infant (8, 9). Rates of screening for PPD in primary care settings are below 50%, indicating missed opportunities to screen more women (10). A common instrument used for screening is the Edinburg Postnatal Depression Scale (EPDS). It is a self-reporting instrument of ten questions that can be completed in minutes and that has been shown to have good reliability (11). Even after diagnosis, it has been reported that only about 13% of PPD cases receive adequate treatment (12). Many women do not receive treatment due to difficulty accessing mental healthcare resources (examples: limited insurance coverage, lack of transportation, lack of childcare) (13). Others do not receive treatment due to social stigmas; for example, fear of being seen as an unfit parent or even losing custody of their child can keep women from the important step of disclosing their mental health struggles (13).

### Role of Digital Technologies in Peripartum Depression Management

One possible solution to bridge inadequacies in access to PPD care is the use of digital technologies and analytics to better understand women's needs and develop responsive solutions to help prevent and self-manage PPD. For example, a mobile health app dedicated to PPD management can provide women with the flexibility of completing a therapy program at their own pace and wherever they choose (14). Such mobile health solutions have been shown to have good acceptance by both clinical providers and peripartum women (15, 16). In order to adequately assist with the prevention and management of PPD, the information architecture of these technologies should be based on robust assessment of peripartum women's information and technology needs. For example, focus group studies conducted regarding women's experiences with use of technology for their pregnancy and mental health management have revealed women's enthusiastic attitudes regarding use of applications as part of their pregnancy journey, and indicate women's willingness and inclination to receive information about mental health and pregnancy from trustworthy sources such as their providers. However, more investigation is needed to thoroughly understand women's information and technology needs in the management of their mental health during the peripartum period. While several studies have conducted focus groups and interviews to gain deeper understanding of women's needs (17–19), data from additional sources such as online social media must be leveraged to capture women's views across different settings, given high use of these platforms by women to manage their health in general and specifically during pregnancies (20, 21). The mining of social media can offer researchers many advantages over other traditional methods (i.e., focus groups and interviews) in determining user needs through capture of rich ecological context: (a) by assessing the nature of user interactions in built digital environments, which allows researchers to unpack technology specifications that are more responsive to individual's sociotechnical needs (22), (b) by natural language modeling that reflects users' culture and emotional context (23), and (c) by harvesting data from an environment that is less controlled than focus groups and interviews, and where participants may feel more enticed to disclose honest opinions (24). However, the inherent representational bias associated with these platforms requires researchers to be cognizant of demographic constituents and community compositions to ensure generalizability of the lessons learned from these analyses and should often complement with alternative traditional methods of inquiry (25). Despite these shortcomings, online forums provide us with unique opportunities to model information needs as well as psychosocial factors related to PPD prevention and management (26).

#### Online Forums and Peripartum Depression Management

Online forums are widely used by women to discuss and obtain information about pregnancy. For example, pregnancy is the number one topic being discussed by women in the health forums of WebMD and drug review websites including Drugs.com (21). In this setting, women can build a sense of community where they provide each other support of all types: emotional (messages of encouragement), instrumental (tools such as, websites for relaxation techniques), and informational (personal knowledge and experiences) (20, 27, 28). More importantly, some studies have shown that online forums are especially useful in eliminating the social stigma surrounding PPD; this allows women more freedom to disclose their experiences and symptoms (27). Furthermore, peer support through social media can play an important factor in increasing patient adherence to positive health behaviors, indicating that health providers can leverage online social forums to reinforce healthcare plans for PPD management (29).

Online forums can help break down barriers to PPD care by providing participants reassurance in knowing that they are not alone in experiencing postnatal mental illness (20, 27). They can also help women better understand PPD, and reduce their own internal stigma regarding mental illness in the postnatal period. Internal stigma refers to the intrapersonal negative statements a person can have regarding a condition or group of people (27). Because of this reduction in internal stigma, it is common for women to disclose PPD symptoms in an online setting rather than face-to-face (20, 27). However, it is precisely the open nature of forums that can also make some women feel apprehensive. Participant's interactions in online forums can be complex. In a previous survey study (28), participants revealed that a negative aspect of the forums was the dramatic nature of some content, and the possibility of some participants feeling left out. Some participants stated that at times, “popular” forum users (users who are very active and receive a lot of peer responses in the forum) could create a sense of “in-groups,” that is to cause other participants to feel ignored. Therefore, health providers should approach online forums with caution. While they can be a useful tool for purposes such as, social support and symptom disclosure (20, 27), these sources can also present potentially harmful content (28). It has been suggested that mental health providers can use social media (including online forums) as an important source of information and feedback regarding their patients (30), therefore providers should have an active role in their patient's use of these resources.

A unique advantage of social media outlets such as online forums is that their data can be systematically analyzed to produce useful information for identification and management of various diseases, including mental health illnesses. Some examples are the application of computational linguistic analysis to identify Twitter posts where users show signs of schizophrenia (31), and the application of machine learning techniques in online cancer support groups to extract participant's behaviors, treatments, and emotions (32). However, within the domain of PPD there are very few existing studies which use systematic methods to analyze content of user posts from social media. In a study using the concept recognition software programs of MetaMapLite (MMLite) and Human Phenotype Ontology (HPO), Chowdhuri and colleagues (33) were able to map PPD terms extracted from 10,584 posts in the online social forums “Postpartum Depression and Postpartum Anxiety Support Group” and “Postpartum Anxiety Support Group” from the commercial website BabyCenter. Some of the most common terms found in the posts were: “anxiety,” “depression,” “insomnia,” “baby,” and “ppd.” Additionally, a mapping of the most common medication names in the posts included “Zoloft,” “Lexapro,” and “Celexa.” An evaluation of MMLite performance in identifying PPD-related terms showed that the software had a precision of 86.7% and a recall of 81.3%. In a similar study, Fatima and colleagues (34) extracted linguistic features from PPD forums in the social media website Reddit. Through use of various machine learning techniques, the researchers were able to predict PPD content in user posts with 86.9% accuracy. This high level of accuracy was achieved by the machine learning model of multilayer perceptron, which outperformed those of logistic regression and support vector machine.

The aforementioned studies on online forums and PPD clearly outline the advantages women can experience from participating in these, as it pertains to the management of their mental health during the peripartum period (i.e., a sense of community, reduced stigma). However, these studies also lack insights on how best to translate the information present in PPD online forums into digital PPD management solutions that women can use in their everyday routines. Therefore, the purpose of this study is to analyze data from online forums through the lens of digital health technology development. We will achieve this by first conducting a qualitative analysis of user post content from two popular PPD online forums. This will provide us with a better understanding of common topics being discussed, and women's PPD-related knowledge, and their accounts of experiences with PPD management. Then, we will develop and apply a machine learning pipeline to automatically label user posts with the major categories of discussion topics. Our findings will ultimately be used to inform the development of a mobile health application dedicated to PPD prevention and management, which is described below.

#### MomMind: A Mobile Health Solution for Peripartum Depression Management

The proposed mobile health application (MomMind) is driven by our digital health development framework *Digilego* (35) and is dedicated to providing education, self-monitoring, activities, and support to women in the peripartum period to help them with reducing risk for PPD or self-management of PPD if already diagnosed. Once the application is set up, users will be able to access six different features that will engage them across multilevel factors (clinical, social) affecting PPD. These features are: (a) “My Diary,” a journaling feature where users can personally narrate their experiences, (b) “MomTalk,” an integrated social forum to share experiences with fellow peripartum women, and receive and provide social support, (c) “My Care,” a bidirectional communication channel with providers, (d) “My Library,” a repository of multimedia educational content, (e) “How am I feeling today?,” a repository of evidence-based surveys (36, 37) to monitor depression symptoms, and (f) “My Tasks,” where users can select tasks meant to alleviate PPD symptoms (e.g., going for a walk, writing a journal entry) from a master task list curated by PPD clinical experts. Users will be able to freely interact with any feature of the application at any time they choose. The application is also meant to facilitate collaboration between users and their provider. For example, through leveraging data from the “My Diary” and “How am I feeling today?” modules, both users and providers can have a better understanding of the user's symptom progress and the underlying reasons behind it. In our previous studies, we employed multiple methods (38, 39) to inform the feature development of the proposed digital platform. In this study, we focus on social media analysis techniques to further advance our development efforts and inform the conception and refinement of empirically-driven digital features.

## Materials and Methods

**Figure 1:**
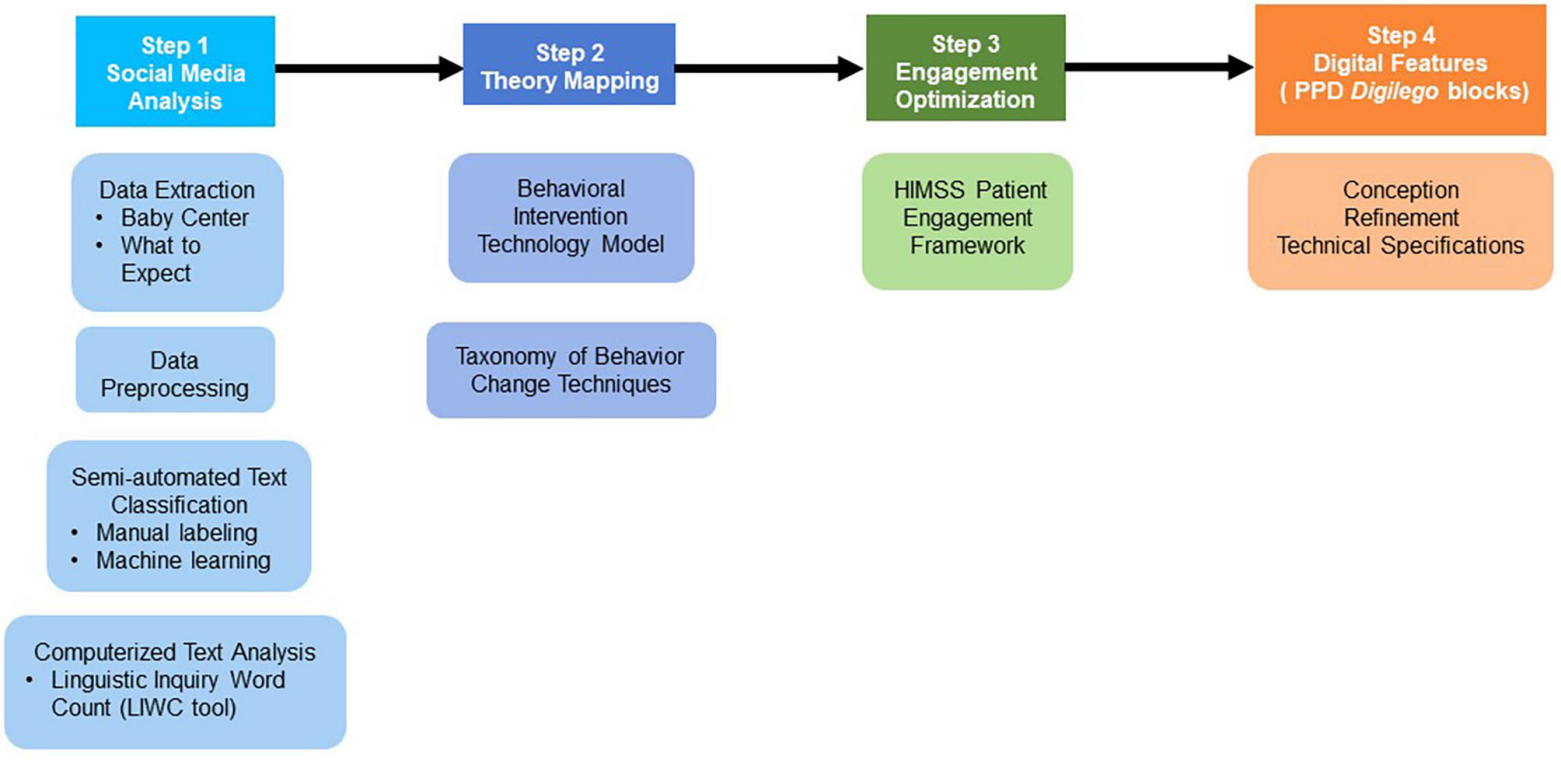
*Digilego*-powered translation: bridging social media analysis to digital health development.

In this study, we use the *Digilego* framework to bridge our social media analysis and digital health development efforts. *Digilego* is a digital health development framework that produces patient-facing digital health solutions that are theory-driven, engaging for sustained use, and that facilitate care coordination processes (35). It is an ideal framework in the context of our study because it includes a component of user needs analysis using mixed methods (ranging from social media to focus groups and interviews). The framework has been previously employed to successfully delineate and implement digital health solutions for young adult cancer survivors by leveraging data from online communities for cancer survivors (40), and it has also been employed in the context of daily stress management (41). Here, we adapt the *Digilego* framework to the specific condition of PPD through the following steps: (1) Social media analysis of PPD online forums, (2) use of Mohr's Behavioral Intervention Technologies (BIT) model as theoretical basis to assist in implementing behavior change, (3) use of the Healthcare Information and Management Systems Society (HIMSS) Patient Engagement Framework (PEF) to ensure optimal user engagement, and (4) development of multi-level and reusable PPD digital features (called *Digilego* blocks) that assist users with various aspects of their care management. Our methods are illustrated in Figure 1.

### Step 1: Social Media Analysis

As part of our user needs analysis, we have created a complete Python pipeline, beginning with the extraction of data from two popular PPD-specific online forums and ending with a final coded dataset containing posts classified by a machine learning (ML) model. All tasks of our pipeline were completed using Python version 3.7 (42). The tasks that we incorporated into this pipeline are: (1) data extraction and pre-processing and (2) Semi-automated multilabel text classification. Because we also wanted to obtain the social and psychological characteristics of user posts, we conducted computerized text analysis using Linguistic Inquiry and Word Count (LIWC) software.

#### Data Extraction and Pre-processing

To acquire the data for our study, we extracted user posts from online forums using web scraping techniques. We used the open-source web-scraping software package Scrapy (43) to accomplish this task. This software allows the user to write their own “spiders.” Spiders are Python programs that crawl websites according to user specifications.

The online forums selected to extract our datasets are:

a) “Postpartum Depression” forum from the website What to Expect (44). What To Expect is based on the book by Heidi Murkoff (45), and it contains forums on a variety of topics. The website receives a total of 800,000 new user posts every month, and has a user population of ~13 million women. The accompanying app is also the best rated pregnancy app in the Apple App store (46).
b) “Postpartum Depression, Anxiety, and Related Topics” from the BabyCenter website (47). BabyCenter is visited by 7 in 10 pregnant and postpartum women who use online resources for information. The website is available in many countries, and its content is available in many languages (48).

The data extracted from the forums is publicly available and has been scrubbed for any identifiers. The text extracted from the online social forums was pre-processed by first removing unwanted characters, white spaces, and making all text lowercase. Then, stopwords (common prepositions such as “a,” “of” which normally do not have semantic meaning) were removed (49). Finally, the process of stemming was applied to each word. Stemming transforms a word into its root format (e.g., “depression” and “depressed” both have the root “depress”) (50). Overall, a dataset with 62,015 posts is considered for analysis.

#### Semi-automated Multilabel Text Classification

##### Manual Coding

To start with, we randomly selected a sub-set of 850 user posts from our extracted dataset of 5,532 posts from What to Expect to perform manual labeling. Coding categories for the user posts, along with examples found in our labeled dataset, are described in Table 1. We arrived at our coding categories through qualitative grounded-theory analysis (51) of our extracted user posts. Open coding was done in a line-by-line analysis of user posts to derive the concepts being discussed. This was followed by axial coding, where patterns and relationships among the open codes were discovered. This process resulted in the emerging of coding categories used in our manual labeling. Our resulting coding categories are not mutually exclusive. Grounded-theory analysis and manual coding was conducted by a single coder. Additionally, a second coder manually coded a randomly selected subset of 100 user posts using the categories derived from grounded theory analysis. This allowed us to assess interrater reliability (Cohen's Kappa). Any discrepancies in manual coding were discussed among both coders until a mutual agreement was reached.

**Table 1:** Coding categories.

**Category**	**Examples**
Family and Friends: content related to interactions with close family members and friends in terms of managing the peripartum period and/or depression symptoms.	“My husband doesn't understand when I become clingy with my baby girl but I feel happier or more at ease when she's in my arms” (Post # 71) “I've talked to my in-laws a couple times, but they're the type to overreact and go too far if they knew I had PPD. They wouldn't be able to leave me alone. So that I'll probably keep to myself. It's just a very frustrating situation.” (Post # 87)
Medications: content related to statements or queries about side effects or others' personal experiences with PPD medications.	“Has anyone started Abilify and had it affect their milk supply?” (Post #116) “Expecting my second baby in the next 2–3 weeks. Had very difficult time with Post-Partum depression the first time and huge delay in getting on Zoloft till dear daughter was 4–5 months. I know it isn't a guarantee to get the second time but curious what others experience once and if any recommends I go on meds shortly after delivery?” (Post # 96)
Symptom Disclosure: a user explicitly describing the depression or anxiety symptoms she/he is experiencing.	“I have anxious spells- with severe symptoms where I feel like I'm in a dream, panicky, heart racing, thoughts that I know I don't actually think. This can go on all day.” (Post #12) “For me it was pretty obvious. A month pp I was crying all the time, disliked my child, was not eating and just felt miserable.” (Post #27)
Social Support: content where a user provides support to their peers. Social support can be of an emotional nature (words of encouragement and kindness), an appraisal (feedback on a situation), an instrument (a practical tool such as a relaxation technique), or informational (educational experiences or resources).	“[…] believe me you are not alone. I think you should get a second opinion honestly. I wish you the best of luck. Maybe try counseling too. Hang in there, you will be great!!!” (Post # 102) “Honestly I think a mommy group or just getting out with family or friends will help you tremendously. Maybe even a therapist. Get out a bit.” (Post # 93)
Breastfeeding: Information on the interactions between mental health, medications, and the breastfeeding process.	“When I stopped breastfeeding it was like all this pressure went away and I felt more relaxed and I could concentrate on getting better.” (Post # 18) “I truly believe that the breastfeeding had a role in it all. I'm still struggling but nowhere near as much as I was while still breastfeeding.” (Post # 14)

#### Multilabel Text Classification

Our study explored the utility of various supervised machine learning (ML) classifiers for automatic text classification. When implementing our ML classifiers, we used a test-train split ratio in our labeled dataset of 0.33–0.67. We also employed the One-vs-the-rest multilabel strategy, in which a binary classifier is trained for each category, and then each category is fitted against all others (52). Through this strategy, each post is coded as 0 or 1 for each label. We evaluated the performance of three ML classifiers using our manually labeled dataset: Logistic Regression (LR), Random Forest (RF), and Support Vector Machine (SVM). Logistic regression functions in text classification tasks by computing the Bayesian probability of a given text falling into a class (53). Random forest classifies text by building a decision tree with subsets of the data (54). SVM performs text classification by separating data into classes after finding the best line of separation between classes. The line of separation can also be called a hyperplane (a two-dimensional space) (55).

The metrics we used for comparison are: precision, recall, and F1 score (56). Precision refers to the fraction of classifications made by the ML model which are correct. Recall refers to the fraction of classifications made by the ML model which coincide with our manual labeling. F1 score is the mean of precision and recall. After model comparison, the final step in our pipeline was to use the best performing model to code a test set of 56,483 previously unseen user posts from BabyCenter.

#### Computerized Text Analysis

We have used LIWC software (57) to obtain social and psychological characteristics of user posts in our manually labeled data set. LIWC breaks down user's writing into basic units like pronouns, and then systematically categorizes words according to pre-defined dictionaries. The main strength of this analysis is that it allows researchers to discover relationships between users' choice words and their psychological characteristics (i.e., how confident, anxious, or angry they are) (58). In our study, we are using LIWC to report the following linguistic dimensions:

a) Average Word Count: the mean number of words per post.
b) Analytic: critical thinking shown in the writing.
c) Clout: the level of confidence in a writer's language.
e) Authenticity: a measure of honesty shown in the writing.
d) Tone: the amount of affective language present in the writing.

The dimensions of Analytic, Clout, Authenticity, and Tone are calculated as the percentage of words which match the words in existing dictionaries representing each dimension. We also explored the emotional characteristics of posts through the following LIWC dimensions: Positive Emotion, Negative Emotion, Anxiety, and Anger. These are also calculated and reported as the percentage of words in a post that match the dictionary words for each emotion dimension.

### Step 2: Theory Mapping

Mohr's Behavioral Intervention Technology (BIT) model (59) helps developers plan the initial stages of technology development by asking five questions: *Why?* (Why are users going to use the technology?), *How?* (How will users reach the established goals of the technology; refers to both conceptual and technical factors), *What?* (What elements will be included in the technology?) and *When?* (When will the users be using the technology?). This offers digital health developers the advantage of planning intervention technology development both at the conceptual and technical levels. We have used this model to advance the initial architecture (developed using focus groups and interviews) of MomMind based on results from our social media analysis.

In this study, the mapping of the *Why, When, and How?* (technical) components of the BIT model to PPD digital features was done in collaboration with a team including two digital health and analytics researchers, as well as two PPD clinical experts. These components were determined through a process of iterative team discussions, in which agreement was reached by all members.

The BIT model is not meant to be used as an exhaustive model in technology development, as some of its components may require other theories or models for further specification of technology development (59). Therefore, to help us answer the conceptual aspect of the BIT model's *How?* question, we have selected Michie's Behavior Change Taxonomy (BCT) (60). This taxonomy will assist us in defining the active ingredients that our technology will employ to facilitate PPD management for the user. The BCT taxonomy is informed by disciplines of psychology, engineering, and behavioral science to create a comprehensive list of 93 possible techniques that are divided into 16 hierarchy groups, ranging from “Goals and planning” to “Covert learning.” These techniques have been previously implemented in cross-domain behavior change interventions and shown to be effective in facilitating a variety of behavior changes for patients. BCTs are useful in promoting effective self-management of disease, and improving patients' engagement in protective health behaviors (61). Because these are two of our main goals with MomMind, the taxonomy is well-aligned with the purposes of our proposed digital health solution. In this study, we have leveraged the results from our social media analysis to choose the best BCTs in the context of PPD self-management through a thorough an iterative mapping process in which a single researcher manually reviewed the complete range of 93 techniques available, and ascertained the utility and scalability of the techniques that best fit to PPD management.

To assist us in outlining the *What?* component of the BIT model, we have employed the Health Information and Management System Society's (HIMSS) Patient Engagement Framework (62) to define the technical elements to be included in MomMind. This is explained in detail in the following Step 3.

### Step 3: Engagement Optimization

The HIMSS PEF framework (62) consists of five cumulative patient engagement levels: “Inform Me,” “Engage Me,” “Empower Me,” “Partner with Me,” and “Support my e-Community.” Within these levels are 14 engagement categories that specify tools to facilitate each engagement level. The level of “Inform Me” begins with the following four engagement categories: Information and Way Finding (i.e., service directory), e-Tools (i.e., health encyclopedia), Forms, and Patient-specific Education. The “Engage Me” and “Empower Me” levels add the engagement categories of Patient Access to Records, Patient-Generated Data, and Interoperable Records. The “Partner with Me” level adds the Collaborative Care engagement category, which allow the patient to access care information from as many specialties as needed. Finally, the “Support My e-Community” level adds the Community Support category, which includes tools such as online forums. We have chosen the framework to identify the digital tools that will help us reach an optimal level of patient engagement in their PPD care, given its granular focus on engaging patients through digital formats (i.e., telehealth visits, electronic patient forms). Additionally, the HIMSS framework is appropriate for engaging the PPD population due to its focus on interdisciplinary clinical collaboration and community building, both important factors in PPD management (63). Our mapping process of the HIMSS PEF framework to digital engagement features for PPD care was similar to our theory mapping described in Step 2: the 14 engagement categories were manually reviewed, and based on the information resulting from our grounded theory analysis, those that best corresponded to our user needs were selected.

### Step 4: Digital Features (PPD-Specific *Digilego* Blocks)

Based on results from our user needs analysis from social media, theory mapping, and engagement optimization, we have described digital features that are the best fit for the technology and information needs of women during the challenging peripartum period. These features are aimed to assist women with the complex task of managing their mental health while meeting family and work responsibilities as a new or expectant mother.

## Results

### Social Media Analysis

#### Data Extraction

We successfully extracted 5,532 user posts from the What to Expect “Postpartum Depression” forum and 56,483 user posts from Baby Center's “Postpartum Depression, Anxiety, and Related Topics” forum. Our dataset was extracted into a CSV format, and contains the following variables: post title, author (online alias), post content, date of post, and number of reply comments for each post.

#### Semi-automated Multilabel Text Classification

##### Manual Coding

**Figure 2:**
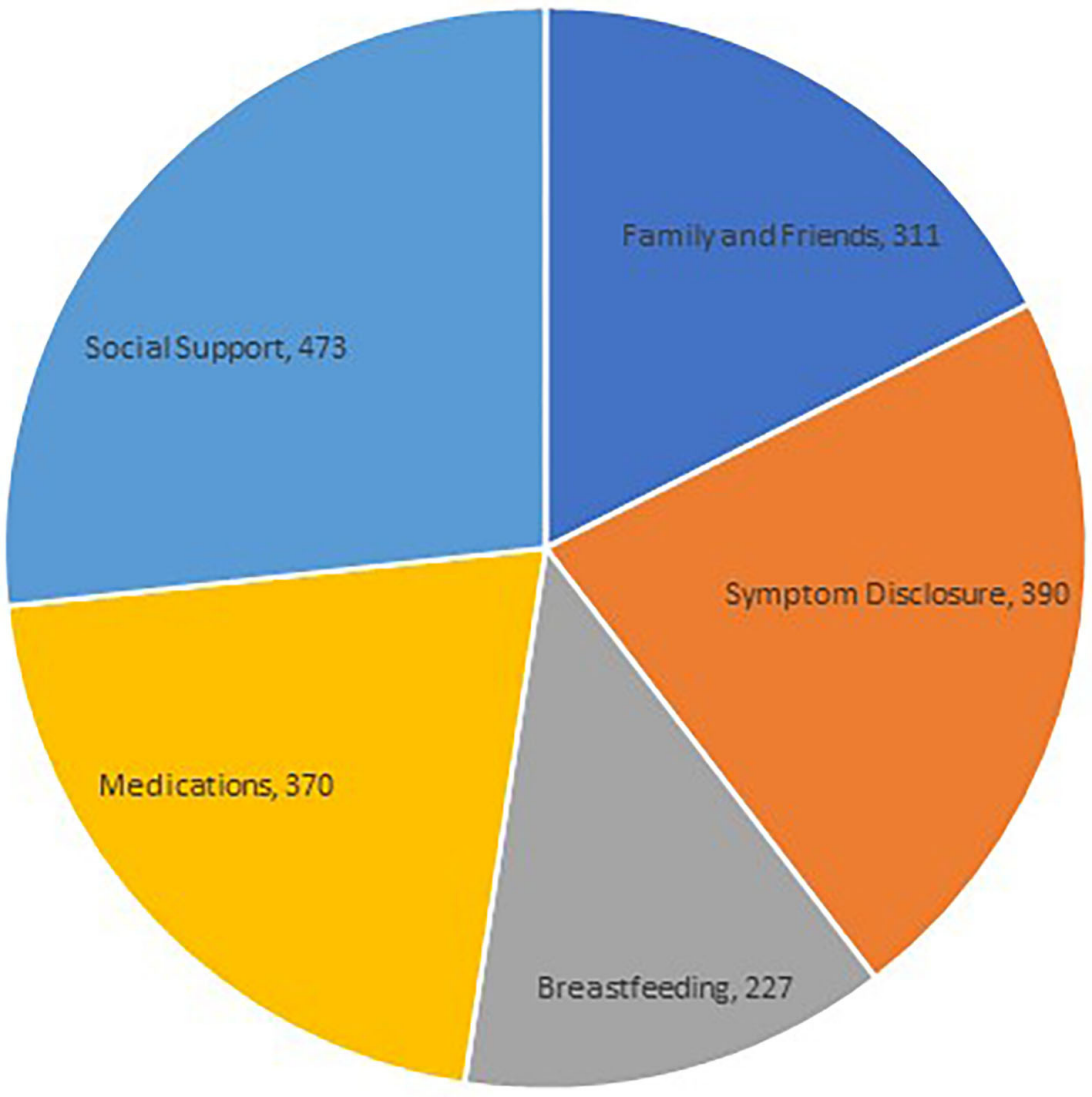
Thematic distribution of PPD-related online social interactions using manual coding.

We randomly selected 850 user posts from our What to Expect extracted dataset for manual labeling. In our manual labeling of user posts, we found that the top category was “Social Support” with 473 posts, followed by “Symptom Disclosure” with 390. The category of “Medications” was applied to 370 posts, and “Family and Friends” to 311 posts. The least applied category was “Breastfeeding” with 227 posts. These results are illustrated in Figure 2. Our interrater reliability assessment indicated that raters had substantial agreement within all categories (Table 2).

**Table 2:** Interrater reliability for manual coding.

**Category**	**Cohen's Kappa**
Family and friends	0.93
Medications	0.89
Symptom disclosure	0.80
Social support	0.79
Breastfeeding	1.0

#### Multilabel Text Classification

**Figure 3:**
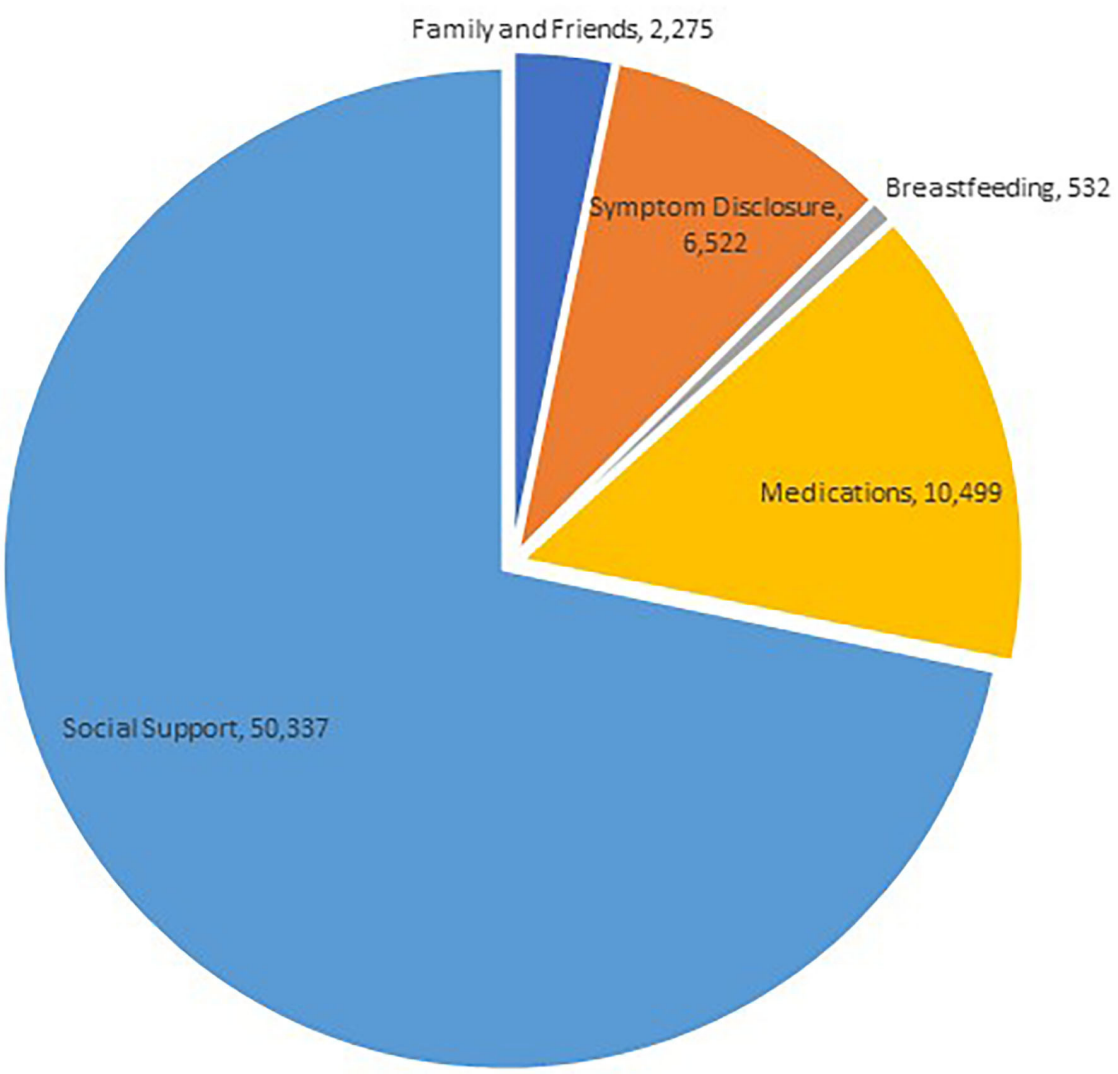
Thematic distributions of PPD-related online social interactions using automated classification.

When comparing the performances of the LR, RF, and SVM models in predicting categories for our labeled data, we found that RF had the best performance. Model performances are summarized in Table 3. We selected RF as the best performing model and used it to predict categories for user comments in our unlabeled dataset of 56,484 user posts from BabyCenter. The most populous category was “Social Support” with 50,337 comments, followed by “Medications” with 10,499. The least used category was “Breastfeeding” with 532 comments. These results are summarized in Figure 3.

**Table 3:** Automatic text classification model performance.

**Category**	**Model**								
	**Logistic regression**			**Random forest**			**Support vector machine**		
	**Precision**	**Recall**	**F1 score**	**Precision**	**Recall**	**F1 score**	**Precision**	**Recall**	**F1 score**
Family and friends	0.89	0.59	0.71	0.86	0.67	0.75	0.80	0.77	0.78
Medications	0.95	0.73	0.83	0.93	0.83	0.88	0.88	0.78	0.83
Symptom disclosure	0.71	0.68	0.69	0.72	0.70	0.71	0.67	0.65	0.66
Social support	0.62	0.85	0.72	0.64	0.83	0.73	0.68	0.72	0.70
Breastfeeding	1.00	0.26	0.41	0.98	0.66	0.79	0.98	0.56	0.71

#### Computerized Text Analysis

Text analysis results using LIWC are reported in Tables 4, 5. A user post contained, on average, 94 words. The average analytic score was 28.31. The average clout score of 44.91 indicates that users were moderately confident within their writing. This is coherent with the nature of online social forum discussions: users are confident of the information they provide because it is based on their personal experiences, yet many are also looking for reassurance or further information on a topic. User posts had an average authenticity score of 65.71. Finally, the average tone score of 39.52 indicates that while many of the posts do consist of emotional content, most of them also show objective content. In regards to the emotional characteristics of the dataset, we found that positive emotions had the highest percentage of words in user posts (Table 5). However, negative emotions did not fall far behind. Anxiety had a very low score, as did Anger. This may be indicative of the supportive nature of online PPD forums.

**Table 4:** Linguistic characteristics of training dataset.

**Dimension**	**Average score**
Word count	94.67
Analytic	28.31
Clout	44.91
Authenticity	65.71
Tone	39.52

**Table 5:** Emotional characteristics of training dataset.

**Dimension**	**Average score**
Positive emotion	4.09
Negative emotion	3.47
Anxiety	1.07
Anger	0.35

Our computerized text analysis helps inform our digital health development process by illustrating the emotionally complex nature of interactions within PPD online forums. Even though these forums are meant to be supportive in nature, at times participants can also exhibit language that is emotionally negative, as shown in the following example:

“*I'm unhappy with my life. I love my daughter. I love my husband most of the time. I hate my step son. I work hard am educated and I bring home a decent salary. I hate the house I live in. Husband bought it with wife 1. It's small and ugly. It's always dirty. I clean and clean and can hardly keep up. We have too many pets to take care of but don't want to get rid of them - they cause a lot of the mess and stress. My husband and crappy step son cause the rest of the mess. I just need a pity party. I want to have that little family I love - without a step kid in it. I want a nice and clean house to come home too. Is that so much to ask*” (Post #691).

### Theory Mapping

Table 6 shows our mapping of the BIT model to the development of technology for the management of PPD. Through this mapping, our collaborating team of PPD clinical experts and digital health and analytics researchers have determined that the main reason behind our technology (the *Why?*) is the increase of women's knowledge about PPD and their subsequently improved self-management of mental health throughout the peripartum period. It was also determined that the technology should take the form of a mobile health application (the technical *How?*), and that women should be able to use it at any desired time during the peripartum period (the *When?*).

**Table 6:** Theory mapping.

**BIT component**	**Mapping to PPD (Examples)**
Why	Increasing PPD knowledge and self-management skills according to user needs analysis, as derived from online PPD-specific social forums.
How (conceptual)	Behavior changes derived from the Taxonomy of Behavior Change Techniques (27)
What	Digital features that facilitate: (a) Medication management. (b) Social support (c) Symptom disclosure (d) Breastfeeding (d) Family and friend dynamics
How (Technical)	Mobile health application
When	Participants will be able to use digital features throughout the peripartum period, and as desired in their daily routines.

The conceptual *How?* of our technology are behavior change techniques appropriate to PPD self-management. These techniques are selected from the existing BCT model and can be seen in further detail in Table 7. All of our selected techniques are responsive to the PPD topics discussed by our target population, as manifested in their social media interactions. For instance, breastfeeding management was an important topic to forum participants. The techniques of associative learning and credible source were selected with this topic in consideration. The *What?* of our technology are digital features that facilitate PPD management. Similar to the conceptual *How?* of our technology, we have defined these features based on the information needs of our target population as shown in the major PPD topics discussed. Therefore, our digital features will facilitate: medication management, social support, symptom disclosure, breastfeeding, and family and friend dynamics. The specific technical elements through which these features will engage our users are listed in more detail in our engagement optimization results (Table 7).

**Table 7:** Mapping of PPD digital features to behavior change techniques and engagement optimization.

**PPD online forum-based user need**	**Example of related digital features**	**BCT techniques**	**HIMSS PEF engagement level (s), engagement category, and related engagement features**
Medications	- Medication list - Medication Instructions - Medication reminders	- Pharmacological Support - Action planning - Self-monitoring of behavior - Instruction on how to perform the behavior	Level: Partner with Me; Category: Patient-Generated Data; Features: Adherence reporting (medications)
Breastfeeding	- Educational videos about breastfeeding and mental health in the peripartum period - Resource information (i.e., contact to lactation consultant)	- Associative learning - Credible source	Level: Inform Me; Category: eTools; Features: Wellness guidance, prevention.
Social support	- Discussion forums	- Social support (unspecified)	Level: Support my e-Community; Category: Community Support; Features: Online Community Support Forums
Symptom disclosure	- Journaling feature - Discussion forums	- Self-talk - Social support (unspecified) - Framing/reframing	Levels: Empower Me, Support my e-Community; Categories: Patient Generated Data; Features: Self-management diaries, Symptoms Assessment.
Family and friends	- “Share” button - “Add user” feature	- Monitoring of behavior by others without feedback- Feedback on behavior - Social support (unspecified)- Social incentive	Level: Support my e-Community; Category: Community Support; Features: Online community support forums and resources for all care team members (caregivers, family, friends)

### Engagement Optimization

Our results for Engagement Optimization are reported in Table 7. Similar to our theory mapping step, our engagement features are based on themes found through our user need analysis. For example, the theme of breastfeeding management was mapped to the lowest patient engagement level in PEF (“Inform Me”) and falls under the engagement category of eTools. The specific engagement tools that are appropriate for this theme are wellness guidance and prevention (which are planned to be implemented in the form of education videos and information resources). The themes of social support and family and friends reach the highest level of patient engagement in PEF (“Support my e-Community”). These themes fall under the engagement category of community support, and features such as online community support forums and resources for all care team members (caregivers, family, friends) are appropriate to address these themes of user need. Table 7 also includes the specific behavior change techniques we have selected for implementation in our technology; these range from pharmacological support to self-talk. The array of patient engagement features and behavior change techniques we have chosen based on our user needs analysis (Table 7) are aligned our intentions with Mom Mind, which are to provide education, self-monitoring, activities, and support to women in the peripartum period.

### Digital Features (*Digilego* Blocks)

Based on results from our previous steps, we have identified the following digital features as appropriate for PPD management:

(a) Medication Management Features: our platform should include features that aid users with medication management and education. The education materials should reinforce that antidepressants, unlike some other medications, are not “one-size fits all,” and that every woman will have unique medication regimes in their mental health care. Educational videos could be included to inform the user of popular antidepressants used during pregnancy, the possible side effects they can experience, and how their doctor will likely be managing their medication (i.e., tapering, combining antidepressants with other medications). Another feature that should be included is a calendar with medication reminders.
(b) Breastfeeding Management Features: It was clear from our content analysis that our users saw a correlation between breastfeeding and their mental health status; interestingly, it seems that most of our users experienced a sense of relief once they stopped breastfeeding. Therefore, our digital platform should include evidenced-based information on the relationship between breastfeeding and PPD as part of the education materials. Ideal education materials would be videos featuring PPD clinical experts explaining different approaches to breastfeeding for women who experience PPD, or an article highlighting recommendations from entities such as the American Academy of Pediatrics or the American College of Obstetricians and Gynecologists. Additionally, our platform can include practical resources such as contact information for lactation consultants.
(c) Social Media Features: Our results clearly indicate that social media provides users with a sense of community and social support. In our content analysis we observed that this sense of community helped users feel like they were not alone in their mental health struggles. This, in turn, helped them with the important step of disclosing their symptoms. Therefore, our platform should include features such as, the ability to share posts and pictures with others, which are common features of social media platforms. However, based on previous work and our qualitative post content analysis, we would not include features that would lead to the platform becoming a popularity contest. These include the features of “Like” and “Follow” buttons. Participants and forum moderators should be able to mark posts of such nature if they contain language that can potentially trigger depression symptoms for other participants or if the post exhibits abusive language. Therefore, features along the lines of a “Warning: Potential Triggers” banner or a “Report User” button should be included in PPD-specific technology to help censor such content.
(d) Shared Access Features: We have observed in our content analysis that most users would like their partners to have a more active role during the peripartum period, but many struggled with involving them in their everyday routine (i.e., caring for baby, household chores) and did not know how to talk to their partners about mental health. Therefore, a feature where the user could provide access to their partner, or friends and family members, to be a participant in the digital platform would be a welcome addition. This would improve communication between the user and those closest to her, and could potentially help these parties notice if the user is experiencing mental health struggles.

## Discussion

Peripartum depression is a condition where social stigmas and difficulty accessing care can prevent women from receiving appropriate treatment and overcome the condition. Therefore, it is a condition that can greatly benefit from digital health interventions. In order to outline the most effective interventions, we have analyzed user needs and sentiments regarding PPD by employing social media analytics methods. We selected two popular online forums, What to Expect and Baby Center, as the settings from which we extracted our data sources. These provided us with extensive insights into what their numerous participants are talking about and feeling regarding PPD.

Our results indicate that our analysis methods were particularly useful in identifying user comments which mention Social Support, as this was one of the themes which was most brought up in the forums. Topics within this theme include: messages of emotional support, advice for improving dynamics with family and friends throughout pregnancy, and arrival of the infant, and practical advice for alleviating PPD symptoms. The second most identified theme was Medications, which indicates that participants of a PPD online forum are apt to discuss personal experiences regarding the medications they have taken (i.e., side effects, dosage). Our results yielded similar themes grounded in social support to those of previous studies about PPD and online forums (27). Our computerized text analysis helped us better identify user's psychological characteristics and sentiments as exhibited in their writing. This analysis revealed that PPD forums are a place where women share a plethora of information, knowledge, and emotions. While most user posts were supportive and objective, some exhibited language that can be considered too explicit. Overall, our social media analysis helped us outline practical digital features that should be optimal in supporting women who experience difficulties navigating their mental health during pregnancy or the newborn stage. These are focused on the specific tasks of medication management and breastfeeding, and they also consider the importance of providing women with social support and incorporating friends and family into their support system. Finally, we also discovered that PPD requires specific digital features for the monitoring and management of women's conversations (i.e., censoring and warning buttons) on what can be a highly sensitive topic.

Our study was presented with some challenges and limitations. The first limitation in our study is that our selected online forums do not contain data such as participant's demographics (age, gender, race, geographic region), or whether they presently have a PPD clinical diagnosis, which prevents an analysis of the correlation between such data and user posts. Additionally, while in this study we observed that online forums provide a sense of community where women can freely discuss their experiences with PPD, we also recognize that such information may not be exhaustive and can be inherently biased (25) and, therefore, should be supplemented with complementary methods of inquiry. For this reason, we conducted our analysis of online PPD forums in conjunction with other data sources such as face-to-face interviews and focus groups as reported in (38, 39). Other limitations of our study include that some aspects of our theory mapping have been conducted by a single researcher. However, our proposed digital health solution for PPD is being developed using human-centered design processes, and therefore incorporates iterative and formative evaluations to ensure the acceptance, feasibility, and usability of these digital features. The next steps in our research program are: (a) to further strengthen our PPD needs analysis by obtaining clinician's perspectives regarding technology use for PPD management, and (b) to develop prototypes that implement the PPD digital features outlined from our user needs analysis.

## Conclusion

Data produced from social media posts offers great opportunities in the areas of mental health management. Currently, there is scarce research work which has leveraged data from online forums exclusively dedicated to the condition of PPD. This data can offer unique insights into how women manage their mental health during the peripartum period, as online forums are a unique setting where some women can feel more comfortable disclosing their stories than in a face-to-face setting. In this study, we have adapted the digital health framework *Digilego* to leverage such data, in order to map digital features that are optimal for engaging peripartum women and implementing behavior changes (i.e., improved medication management, improved family communication) that will make their transition into motherhood a smoother process. Through the combination of robust social media analysis methods and mapping to existing behavior change and engagement models, our resulting digital features are equipped to meet the information and technology needs of peripartum women. In our study, we have found that the most important themes being discussed in online PPD forums were: medications, breastfeeding, symptom disclosure, family and friends, and social support. The ability to add partners or family and friends as participants in a digital platform, and the ability to learn how to manage practical aspects of the peripartum period (breastfeeding, medications) would be acceptable digital features to our users. One of the strengths of our study is that our social media data analysis guided our technology development in conjunction with data from focus groups, providing us a more thorough view of women's information and technology needs while managing their mental health during the complex peripartum period.

This work supports findings from previous studies that recommend the development of affordable, scalable technologies which capture a thorough view of women's pregnancy journeys. Such technologies will help PPD stakeholders (doctors, perinatal women, family members) better detect changes in women's mental health status and offer assistance at early stages (64, 65). This approach has been previously used successfully to promote PPD screening (66). Similar to other studies (67), this analysis revealed how perinatal women use social media as a resource for practical purposes such as, breastfeeding, and it highlights the importance of using social media data to produce evidence-based patient engagement tools. Indeed, social media channels have been used to assist perinatal women with various aspects of having a new baby, such as healthy infant weight management i.e., (68). However, as also suggested in other studies, more women are resorting to their smartphones for information seeking and social support sources (69). Therefore, in this study we want to leverage our social media analysis to a platform that is not only web-based, but rather an application that women can access from their smartphones at any time and any place and facilitates multilevel interventional points. This application would provide not only peer support, but also PPD education and bidirectional communication with clinicians. This is in line with previous research that shows perinatal women have unique information and emotional needs at different stages of pregnancy, therefore social media tools should be expanded to include a wide range of resources (70).

## Data Availability Statement

The raw data supporting the conclusions of this article will be made available by the authors, without undue reservation.

## Author Contributions

All authors listed have made a substantial, direct and intellectual contribution to the work, and approved it for publication.

## Conflict of Interest

The authors declare that the research was conducted in the absence of any commercial or financial relationships that could be construed as a potential conflict of interest.

## References

[1] Vigod SN Wilson CA Howard LM. Depression in pregnancy. BMJ. (2016) 352:i1547. 10.1136/bmj.i1547 27013603

[2] Ko JY Rockhill KM Tong VT Morrow B Farr SL. Trends in postpartum depressive symptoms — 27 states, 2004, 2008, and 2012. MMWR Morb Mortal Wkly Rep. (2017) 66:153–8. 10.15585/mmwr.mm6606a1 28207685 PMC5657855

[3] Hübner-Liebermann B Hausner H Wittmann M. Recognizing and treating peripartum depression. Dtsch Arztebl Int. (2012) 109:419–24. 10.3238/arztebl.2012.0419 22787503 PMC3394379

[4] Centers for Disease Control and Prevention. Depression among Women. Atlanta, GA: Centers for Disease Control and Prevention (2020). Available online at: https://www.cdc.gov/reproductivehealth/depression/index.htm (accessed April 8, 2020).

[5] Righetti-Veltema M Conne-Perréard E Bousquet A Manzano J. Risk factors and predictive signs of postpartum depression. J Affect Disord. (1998) 49:167–80. 10.1016/S0165-0327(97)00110-9 9629946

[6] Fitelson E Kim S Baker AS Leight K. Treatment of postpartum depression: clinical, psychological and pharmacological options. Int J Womens Health. (2010) 3:1–14. 10.2147/IJWH.S6938 21339932 PMC3039003

[7] Anxiety and Depression Association of America. Postpartum Depression. Silver Spring, MD: Anxiety and Depression Association of America (2018). Available online at: https://adaa.org/living-with-anxiety/women/postpartum-depression (accessed April 8, 2020).

[8] Field T. Postpartum depression effects on early interactions, parenting, and safety practices: a review. Infant Behav Dev. (2010) 33:1–6. 10.1016/j.infbeh.2009.10.005 19962196 PMC2819576

[9] Mayo Clinic. Peripartum Depression. (2020). Available online at: https://www.mayoclinic.org/diseases-conditions/postpartum-depression/symptoms-causes/syc-20376617 (accessed January 7, 2021).

[10] Gjerdingen DK Yawn BP. Postpartum depression screening: importance, methods, barriers, and recommendations for practice. J Am Board Fam Med. (2007) 20:280–8. 10.3122/jabfm.2007.03.060171 17478661

[11] McBride HL Wiens RM McDonald MJ Cox DW Chan EK. The Edinburgh Postnatal Depression Scale (EPDS): a review of the reported validity evidence. In: Zumbo BD Chan EKH, editors. Validity and Validation in Social, Behavioral, and Health Sciences. Cham: Springer (2014). p. 157–74. 10.1007/978-3-319-07794-9_9

[12] Cox EQ Sowa NA Meltzer-Brody SE Gaynes BN. The perinatal depression treatment cascade: baby steps toward improving outcomes. J Clin Psychiatry. (2016) 77:1189–2000. 10.4088/JCP.15r10174 27780317

[13] Grimbergen A Raghuram A Dorland JM Miller CC Correa N Bocchini C. Perinatal depression policy brief:25. Available online at: https://www.texaschildrens.org/sites/default/files/uploads/documents/77113_Pages_PolicyBrief_PPD.pdf (accessed January 10, 2021).

[14] Hollis C Morriss R Martin J. Technological innovations in mental healthcare: harnessing the digital revolution. Br J Psychiatry. (2015) 206:263–5. 10.1192/bjp.bp.113.142612 25833865

[15] Osma J Barrera A Ramphos E. Are pregnant and postpartum women interested in health-related apps? Implications for the prevention of perinatal depression. Cyberpsychol Behav Soc Netw. (2016) 19:412–5. 10.1089/cyber.2015.0549 27327069

[16] Sprenger M Mettler T Osma J. Health professionals' perspective on the promotion of e-mental health apps in the context of maternal depression. PLoS ONE. (2017) 12:e0180867. 10.1371/journal.pone.0180867 28704442 PMC5507525

[17] Doherty K Barry M Marcano-Belisario J Arnaud B Morrison C Car J. A mobile app for the self-report of psychological well-being during pregnancy (brightself): qualitative design study. JMIR Ment Health. (2018) 5:e10007. 10.2196/10007 30482742 PMC6290271

[18] Bhat A Mao J Unützer J Reed S Unger J. Text messaging to support a perinatal collaborative care model for depression: a multi-methods inquiry. Gen Hosp Psychiatry. (2018) 52:14–20. 10.1016/j.genhosppsych.2018.01.005 29494854 PMC5936469

[19] Bhat A Reed S Mao J Vredevoogd M Russo J Unger J. Delivering perinatal depression care in a rural obstetric setting: a mixed methods study of feasibility, acceptability and effectiveness. J Psychosom Obstet Gynaecol. (2018) 39:273–80. 10.1080/0167482X.2017.1367381 28882096 PMC6203656

[20] Moore D Drey N Ayers S. Use of online forums for perinatal mental illness, stigma, and disclosure: an exploratory model. JMIR Ment Health. (2017) 4:e6. 10.2196/mental.5926 28219879 PMC5339438

[21] Sadah SA Shahbazi M Wiley MT Hristidis V. Demographic-based content analysis of web-based health-related social media. J Med Internet Res. (2016) 18:e148. 10.2196/jmir.5327 27296242 PMC4923586

[22] Oser TK Oser SM Parascando JA Hessler-Jones D Sciamanna CN Sparling K. Social media in the diabetes community: a novel way to assess psychosocial needs in people with diabetes and their caregivers. Curr Diab Rep. (2020) 20:10. 10.1007/s11892-020-1294-3 32080765

[23] Myneni S Fujimoto K Cohen T. Leveraging social media for health promotion and behavior change: methods of analysis and opportunities for intervention. In: Patel VL Arocha JF Ancker JS, editors. Cognitive Informatics in Health and Biomedicine: Understanding and Modeling Health Behaviors. Cham: Springer International Publishing (2017). p. 315–45. 10.1007/978-3-319-51732-2_15

[24] Shadpour D. Council Post: How Social Media can Serve as the New Focus Group for Your Brand. Forbes. Available online at: https://www.forbes.com/sites/forbesagencycouncil/2018/03/21/how-social-media-can-serve-as-the-new-focus-group-for-your-brand/ (accessed Febraury 17, 2021).

[25] Olteanu A Castillo C Diaz F Kiciman E. Social data: biases, methodological pitfalls, and ethical boundaries. Front Big Data. (2019) 2:13. 10.3389/fdata.2019.00013 33693336 PMC7931947

[26] Singh T Roberts K Cohen T Cobb N Wang J Fujimoto K. Social media as a research tool (SMaaRT) for risky behavior analytics: methodological review. JMIR Public Health Surveill. (2020) 6:e21660. 10.2196/21660 33252345 PMC7735906

[27] Moore D Ayers S. Virtual voices: social support and stigma in postnatal mental illness internet forums. Psychol Health Med. (2017) 22:546–51. 10.1080/13548506.2016.1189580 27218265

[28] Teaford D McNiesh S Goyal D. New mothers' experiences with online postpartum forums. MCN Am J Matern Child Nurs. (2019) 44:40–5. 10.1097/NMC.0000000000000489 30444739

[29] Lin RJ Zhu X. Leveraging social media for preventive care-A gamification system and insights. Stud Health Technol Inform. (2012) 180:838–42. 22874310

[30] Shepherd A Sanders C Doyle M Shaw J. Using social media for support and feedback by mental health service users: thematic analysis of a twitter conversation. BMC psychiatry. (2015) 15:29. 10.1186/s12888-015-0408-y 25881089 PMC4337200

[31] Birnbaum ML Ernala SK Rizvi AF De Choudhury M Kane JM. A collaborative approach to identifying social media markers of schizophrenia by employing machine learning and clinical appraisals. J Med Internet Res. (2017) 19:e289. 10.2196/jmir.7956 28807891 PMC5575421

[32] De Silva D Ranasinghe W Bandaragoda T Adikari A Mills N Iddamalgoda L. Machine learning to support social media empowered patients in cancer care and cancer treatment decisions. PLoS ONE. (2018) 13:e0205855. 10.1371/journal.pone.0205855 30335805 PMC6193663

[33] Chowdhuri S McCrea S Fushman DD Taylor CO. Extracting biomedical terms from postpartum depression online health communities. AMIA Jt Summits Transl Sci Proc. (2019) 2019:592–601. 31259014 PMC6568056

[34] Fatima I Abbasi BU Khan S Al-Saeed M Ahmad HF Mumtaz R. Prediction of postpartum depression using machine learning techniques from social media text. Expert Systems. (2019) 36:e12409. 10.1111/exsy.12409

[35] Myneni S Rogith D Franklin A. Digilego: a standardized analytics-driven consumer-oriented connected health framework. In: International Conference on Social Computing, Behavioral-Cultural Modeling and Prediction and Behavior Representation in Modeling and Simulation. Cham: Springer (2018). p. 263–73. 10.1007/978-3-319-93372-6_30

[36] Delft Institute of Positive Design. Pick-A-Mood Pictorial Tool for Mood Measurement. Available online at: https://diopd.org/pick-a-mood/ (accessed March 14, 2021).

[37] Kroenke K Spitzer RL Williams JB. The patient health questionnaire-2: validity of a two-item depression screener. Med Care. (2003) 41:1284–92. 10.1097/01.MLR.0000093487.78664.3C 14583691

[38] Zingg A Rogith D Refuerzo J Myneni S. Digilego for peripartum depression: a novel patient-facing digital health instantiation. In: Proceedings of the American Medical Informatics Association. (2020). Virtual Annual Symposium, Virtual Annual Symposium Digital Collection, AMIA (2020). Available from: https://s4.goeshow.com/amia/annual/2020/index.cfm (accessed January 12, 2021). PMC8075464 33936518

[39] Zingg A Carter L Rogith D Franklin A Selvaraj S Refuerzo J. Digital technology needs in maternal mental health: a qualitative inquiry. In: 31st Medical Informatics Europe Conference. Athens, (2021). 10.3233/SHTI210324 34042819

[40] Myneni S Amith M Geng Y Tao C. Towards an ontology-driven framework to enable development of personalized mHealth solutions for Cancer survivors' engagement in healthy living. Stud Health Technol Inform. (2015) 216:113. 26262021 PMC4946640

[41] Carter L Rogith D Franklin A Myneni S. NewCope: a theory-linked mobile application for stress education and management. Stud Health Technol Inform. (2019) 264:1150–4. 10.3233/SHTI190406 31438105 PMC7656960

[42] Python Software Foundation. Welcome to Python.org. https://www.python.org/. Available online at: https://www.python.org/ (accessed December 7, 2020)

[43] Scrapy. A Fast and Powerful Scraping and Web Crawling Framework. Available online at: https://scrapy.org/ (accessed December 7, 2021).

[44] What to Expect. Postpartum Depression. (2020). Available online at: https://community.whattoexpect.com/forums/postpartum-depression.html (accessed April 8, 2020).

[45] Murkoff H. What to Expect When You're Expecting. New York, NY: Workman Publishing (2016).

[46] What to Expect. About What To Expect. (2020). Available online at: https://www.whattoexpect.com/about-what-to-expect/ (accessed April 8, 2020).

[47] BabyCenter. Postpartum Depression and Related Topics. (2020). Available online at: https://community.babycenter.com/groups/a15325 (accessed April 8, 2020).

[48] BabyCenter. Company Information. (2020). Available online at: https://www.babycenter.com/about (accessed April 8, 2020).

[49] Dropping Common Terms: Stop Words. Available online at: https://nlp.stanford.edu/IR-book/html/htmledition/dropping-common-terms-stop-words-1.html (accessed December 7, 2020).

[50] Boyer C Dolamic L Grabar N. Automated detection of health websites' HONcode conformity: can N-gram tokenization replace stemming? Stud Health Technol Inform. (2015) 216:1064. 26262363

[51] Charmaz K Belgrave L. Qualitative interviewing and grounded theory analysis. The SAGE handbook of interview research: The complexity of the craft. Thousand Oaks, CA: SAGE Publications Inc. (2012) p. 347–65. 10.4135/9781452218403.n25

[52] Scikit-Learn Developers (BSD License). sklearn.multiclass.OneVsRestClassifier — scikit-learn 0.23.2 Documentation. Available online at: https://scikit-learn.org/stable/modules/generated/sklearn.multiclass.OneVsRestClassifier.html (accessed December 8, 2020).

[53] Genkin A Lewis DD Madigan D. Large-scale Bayesian logistic regression for text categorization. Technometrics. (2007) 49:291–304. 10.1198/004017007000000245

[54] Breiman L. Random forests. Mach Learn. (2001) 45:5–32. 10.1023/A:1010933404324

[55] Joachims T. Learning to Classify Text Using Support Vector Machines: Methods, Theory and Algorithms (The Springer International Series in Engineering and Computer Science). Boston, MA: Springer (2002). p. 228. 10.1007/978-1-4615-0907-3_3

[56] sklearn.metrics.precision_recall_fscore_support — scikit-learn 0.23.2 Documentation. Available online at: https://scikit-learn.org/stable/modules/generated/sklearn.metrics.precision_recall_fscore_support.html (accessed December 6, 2020).

[57] Pennebaker JW Booth RJ Boyd RL Francis ME. Linguistic Inquiry and Word Count: LIWC2015. Austin, TX: Pennebaker Conglomerates (2015). Available online at: www.LIWC.net.

[58] Tausczik YR Pennebaker JW. The psychological meaning of words: LIWC and computerized text analysis methods. J Lang Soc Psychol. (2010) 29:24–54. 10.1177/0261927X09351676

[59] Mohr DC Schueller SM Montague E Burns MN Rashidi P. The behavioral intervention technology model: an integrated conceptual and technological framework for eHealth and mHealth interventions. J Med Internet Res. (2014) 16:e146. 10.2196/jmir.3077 24905070 PMC4071229

[60] Michie S West R Sheals K Godinho CA. Evaluating the effectiveness of behavior change techniques in health-related behavior: a scoping review of methods used. Transl Behav Med. (2018) 8:212–24. 10.1093/tbm/ibx019 29381786 PMC6062857

[61] Abraham C Michie S. A taxonomy of behavior change techniques used in interventions. Health Psychol. (2008) 27:379. 10.1037/0278-6133.27.3.379 18624603

[62] HIMSS Patient Engagement Framework Center for Patient and Family-Centered Care. (2014). Available online at: https://www.nationalehealth.org/patient-engagement-framework/ (accessed December 11, 2020).

[63] Selix N Henshaw E Barrera A Botcheva L Huie E Kaufman G. Interdisciplinary collaboration in maternal mental health. MCN Am J Matern Child Nurs. (2017) 42:226–31. 10.1097/NMC.0000000000000343 28301335

[64] De Choudhury M Counts S Horvitz EJ Hoff A. Characterizing and predicting postpartum depression from shared facebook data. In: Proceedings of the 17th ACM Conference on Computer Supported Cooperative Work & Social Computing. Baltimore MD: ACM. (2014). p. 626–38. 10.1145/2531602.2531675

[65] De Choudhury M Counts S Horvitz E. Predicting postpartum changes in emotion and behavior via social media. In: Proceedings of the SIGCHI Conference on Human Factors in Computing Systems. Paris: ACM. (2013) p. 3267–76. 10.1145/2470654.2466447

[66] Doherty K Marcano-Belisario J Cohn M Mastellos N Morrison C Car J. Engagement with mental health screening on mobile devices: results from an antenatal feasibility study. In: CHI'19: Proceedings of the SIGCHI Conference on Human Factors in Computing Systems. New York, NY: Association for Computing Machinery (2019). p. 1–15. 10.1145/3290605.3300416

[67] Guerra-Reyes L Christie VM Prabhakar A Harris AL Siek KA. Postpartum health information seeking using mobile phones: experiences of low-income mothers. Matern Child Health J. (2016) 20(Suppl. 1):13–21. 10.1007/s10995-016-2185-8 27639571 PMC5118389

[68] Fiks AG Gruver RS Bishop-Gilyard CT Shults J Virudachalam S Suh AW. A social media peer group for mothers to prevent obesity from infancy: the grow2gether randomized trial. Child Obes. (2017) 13:356–68. 10.1089/chi.2017.0042 28557558 PMC5647509

[69] Hussain-Shamsy N Shah A Vigod SN Zaheer J Seto E. Mobile health for perinatal depression and anxiety: scoping review. J Med Int Res. (2020) 22:e17011. 10.2196/17011 32281939 PMC7186872

[70] Gui X Chen Y Kou Y Pine K Chen Y. Investigating support seeking from peers for pregnancy in online health communities. Proc ACM Hum-Comput Interact. (2017) 1:19. 10.1145/3134685

